# mTORC2/RICTOR exerts differential levels of metabolic control in human embryonic, mesenchymal and neural stem cells

**DOI:** 10.1007/s13238-021-00898-9

**Published:** 2022-01-17

**Authors:** Qun Chu, Feifei Liu, Yifang He, Xiaoyu Jiang, Yusheng Cai, Zeming Wu, Kaowen Yan, Lingling Geng, Yichen Zhang, Huyi Feng, Kaixin Zhou, Si Wang, Weiqi Zhang, Guang-Hui Liu, Shuai Ma, Jing Qu, Moshi Song

**Affiliations:** 1 State Key Laboratory of Stem Cell and Reproductive Biology, Institute of Zoology, Chinese Academy of Sciences, Beijing, 100101 China; 2 State Key Laboratory of Membrane Biology, Institute of Zoology, Chinese Academy of Sciences, Beijing, 100101 China; 3 Institute for Stem Cell and Regeneration, Chinese Academy of Sciences, Beijing, 100101 China; 4 Beijing Institute for Stem Cell and Regenerative Medicine, Beijing, 100101 China; 5 Advanced Innovation Center for Human Brain Protection, National Clinical Research Center for Geriatric Disorders, Xuanwu Hospital Capital Medical University, Beijing, 100053 China; 6 CAS Key Laboratory of Genomic and Precision Medicine, Beijing Institute of Genomics, Chinese Academy of Sciences, Beijing, 100101 China; 7 China National Center for Bioinformation, Beijing, 100101 China; 8 University of Chinese Academy of Sciences, Beijing, 100049 China; 9 Aging Translational Medicine Center, International Center for Aging and Cancer, Xuanwu Hospital, Capital Medical University, Beijing, 100053 China; 10 Sino-Danish College, University of Chinese Academy of Sciences, Beijing, 101408 China; 11 Chongqing Renji Hospital, University of Chinese Academy of Sciences, Chongqing, 400062 China; 12 College of Life Sciences, University of Chinese Academy of Sciences, Beijing, 100049 China

**Dear Editor,**

Stem cells, including pluripotent stem cells and adult stem cells, possess the remarkable capability of being able to self-renew while at the same time having potential to differentiate into different cell lineages and functionally distinct cell types. Human embryonic stem cells (hESCs) can differentiate into all adult stem cell types, including human mesenchymal stem cells (hMSCs) and human neural stem cells (hNSCs), but can also give rise to all terminally differentiated cell types (Wang et al., 2021a). Through the continuous replenishment of differentiated cells, stem cells support tissue homeostasis and respond to tissue injuries. Given the promising applications of stem cells in cell therapy and regenerative medicine, insights into molecular events underlying stem cell maintenance, self-renewal ability and pluripotency, continue to garner strong interest (Shan et al., 2021). Although metabolic pathways have been implicated in the reciprocal regulations of stem cell self-renewal and differentiation as well as organ homeostatic maintenance (Garcia-Prat et al., 2017), central aspects of how metabolic requirements differ and are regulated across the various types of human stem cells in our body remain enigmatic.

The mammalian target of rapamycin (mTOR) pathway is one of the most important metabolic pathways in mammals, and accordingly, it is pivotal for multiple cellular activities in stem cells (Meng et al., 2018). The mTOR pathway comprises two distinct protein complexes, complex 1 (mTORC1) with associated regulatory protein RAPTOR, and complex 2 (mTORC2) with the rapamycin-insensitive companion RICTOR, as respective core unique components. Given that numerous clinical trials have been funded to investigate the safety and efficacy of mTOR inhibitors, it is of both scientific and clinical importance to gain a deeper knowledge about the role of mTOR pathway, and especially for the lesser known mTORC2, in the homeostatic maintenance of different types of human stem cells.

To investigate whether and how RICTOR may regulate human stem cells, we used CRISPR/Cas9-mediated gene editing in hESCs to introduce indels in exon 1 of the *RICTOR* gene (Fig. 1A and 1B). This approach successfully knocked out RICTOR as validated by western blot analysis showing the ablation of RICTOR protein. Alongside, S473 phosphorylation of AKT, which is a major substrate of mTORC2 (Wang et al., 2021b), was also downregulated in *RICTOR*^−/−^ hESCs (Figs. 1C and S1A). RICTOR-deficient hESCs were karyotypically normal (Fig. S1B) and maintained genomic integrity, as exemplified by genome-wide copy number variation (CNV) analysis (Fig. S1C). However, RICTOR deficiency caused aberrant colony morphology (Fig. 1D), shown as a marginal differentiation of the clone, a phenotype frequently observed in hESCs with compromised pluripotency. Concomitantly, the expression of stem cell pluripotency markers OCT4, SOX2 and NANOG, along with others, were downregulated (Figs. 1E, S1D and S1E). Disrupted alkaline phosphatase staining and attenuated capability for teratoma formation further confirmed the impaired pluripotency of *RICTOR*^−/−^ hESCs (Fig. 1F–1H). Additionally, RICTOR deficiency attenuated clonal expansion ability and disrupted hESC cell cycle kinetics (Figs. 1I and S1F). Altogether, these observations suggest that RICTOR deficiency attenuates hESC self-renewal and pluripotency.

**Figure 1:**
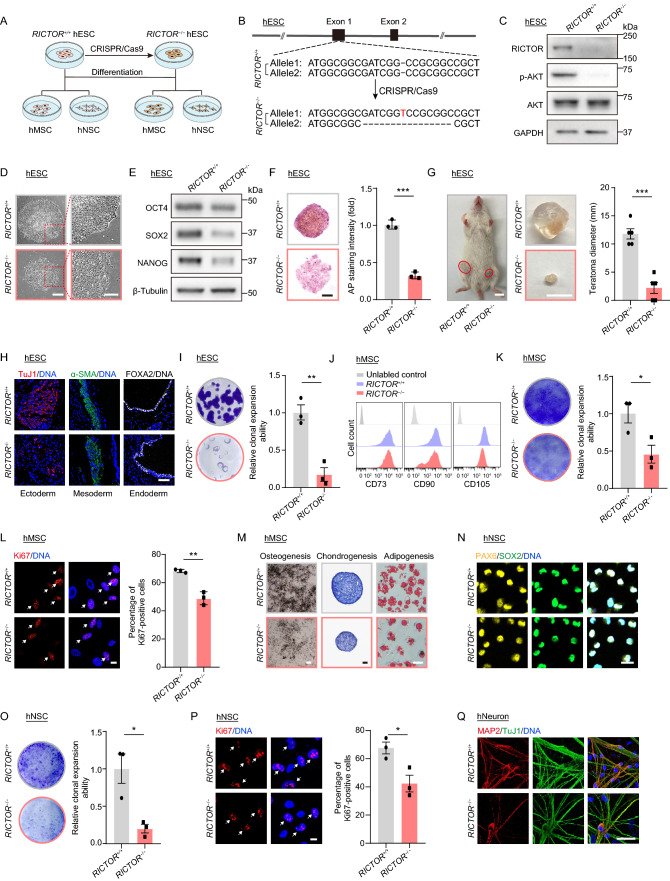
**RICTOR deficiency attenuates pluripotency and differentiation abilities of hESCs, hMSCs and hNSCs.** (A) Schematic diagram of the generation of RICTOR-deficient hESCs and hESC-derived hMSCs and hNSCs. (B) Schematic of the deletion of *RICTOR* via CRISPR/Cas9-mediated non-homologous end-joining (NHEJ). The diagram shows the first 2 out of 38 exons of *RICTOR* along with the edited sequence in exon 1. (C) Western blot analysis of RICTOR, phosphorylated AKT Ser473 and total AKT expression in *RICTOR*^+/+^ and *RICTOR*^−/−^ hESCs. GAPDH was used as the loading control. (D) Representative phase-contrast images of *RICTOR*^+/+^ and *RICTOR*^−/−^ hESC colonies. Scale bar, 200 μm and 100 μm (zoomed-in image). (E) Western blot analysis of the pluripotency markers OCT4, SOX2 and NANOG expression in *RICTOR*^+/+^ and *RICTOR*^−/−^ hESCs. β-Tubulin was used as the loading control. (F) Representative alkaline phosphatase staining of hESCs. Scale bar, 200 μm. Data are representative of three biological repeats. ***P < 0.001. (G) Average diameters of teratomas formed by *RICTOR*^*+/+*^ and *RICTOR*^−/−^ hESCs. Scale bar, 10 mm. Data are presented as mean ± SEM of five biological repeats. ***P < 0.001. (H) Immunofluorescence staining of the differentiation markers for three germ layers in teratoma, Scale bar, 50 µm. (I) Clonal expansion analysis of *RICTOR*^+/+^ and *RICTOR*^−/−^ hESCs. Data are presented as mean ± SEM of three independent experiments. **P < 0.01. (J) Flow cytometry analysis of hMSC-specific surface markers CD73, CD90 and CD105 in *RICTOR*^+/+^ and *RICTOR*^−/−^ hMSCs (passage 3). (K) Clonal expansion analysis of *RICTOR*^*+/+*^ and *RICTOR*^−/−^ hMSCs (passage 4). Data are presented as mean ± SEM of three independent experiments. *P < 0.05. (L) Immunofluorescence analysis of Ki67 expression in *RICTOR*^+/+^ and *RICTOR*^−/−^ hMSCs (passage 4). Scale bar, 10 µm. Data are presented as mean ± SEM of three biological repeats. **P < 0.01. (M) Characterization of the multilineage differentiation potentials of hMSCs (passage 4). Left, osteogenesis of *RICTOR*^+/+^ and *RICTOR*^−/−^ hMSCs evaluated by von Kossa staining. Scale bar, 250 μm. Middle, chondrogenesis of *RICTOR*^+/+^ and *RICTOR*^−/−^ hMSCs evaluated by Toluidine blue staining. Scale bar, 50 μm. Right, adipogenesis of *RICTOR*^+/+^ and *RICTOR*^−/−^ hMSCs evaluated by Oil Red O staining. Scale bar, 50 μm. (N) Immunofluorescence staining for hNSC-specific markers PAX6 and SOX2 in *RICTOR*^+/+^ and *RICTOR*^−/−^ of hNSCs (passage 3). Scale bar, 20 μm. (O) Clonal expansion analysis of *RICTOR*^+/+^ and *RICTOR*^−/−^ hNSCs (passage 5). Data are presented as mean ± SEM of three independent experiments. *P < 0.05. (P) Immunofluorescence analysis of Ki67 expression in *RICTOR*^+/+^ and *RICTOR*^−/−^ hNSCs (passage 5). Scale bar, 10 µm. Data are presented as mean ± SEM of three biological repeats. *P < 0.05. (Q**)** Immunofluorescence staining of hNeuron-specific markers MAP2 and TuJ1 in *RICTOR*^+/+^ and *RICTOR*^−/−^ hNeurons. Scale bar, 50 µm

Next, we differentiated *RICTOR*^+/+^ and *RICTOR*^−/−^ hESCs into hMSCs and hNSCs to assess how RICTOR loss-of-function (LOF) impacts on human adult stem cells (Fig. 1A). As expected, RICTOR protein was depleted in *RICTOR*^−/−^ hMSCs along with decreased AKT S473 phosphorylation (Fig. S1G and S1H). Yet, *RICTOR*^−/−^ hMSCs maintained genomic integrity as demonstrated by CNV analysis (Fig. S1I) and normal cellular morphology along with the expression of hMSC-specific markers including CD73, CD90 and CD105 as previously described (Bi et al., 2020) (Figs. 1J and S1J). By contrast, RICTOR deficiency led to decreased self-renewal ability in hMSCs, as evidenced by lower clonal expansion ability, fewer Ki67 positive cells (Fig. 1K and 1L). RICTOR deficiency also impeded the differentiation potentials of hMSCs into osteoblasts, chondrocytes and adipocytes (Figs. 1M and S1K). As for *RICTOR*^−/−^ hNSCs, RICTOR protein was completely absent together with decreased AKT S473 phosphorylation (Fig. S1L and S1M). Likewise, *RICTOR*^−/−^ hNSCs expressed hNSC-specific markers PAX6 and SOX2 (Figs. 1N and S1N) as previously described (Wang et al., 2020) and maintained genomic integrity, as shown by CNV analysis (Fig. S1O). In line with our findings in hMSCs, RICTOR deficiency led to decreased proliferative ability (Fig. 1O and 1P) and impaired neuronal differentiation in hNSCs, as reflected by lower percentage of cells positive for neuron-specific markers MAP2 (Figs. 1Q and S1P). Altogether, these results indicate that RICTOR deficiency impairs self-renewal and differentiation of hMSCs and hNSCs.

To elucidate molecular mechanisms underlying RICTOR LOF and how these contribute to compromised hESC self-renewal and differentiation capabilities, we carried out genome-wide RNA sequencing (RNA-seq) analysis. In total, we identified 585 upregulated genes and 530 downregulated genes in *RICTOR*^−/−^ hESCs (Fig. S2A and S2B). Pathway and gene ontology (GO) enrichment analysis revealed that the upregulated genes were mainly associated with oxidative phosphorylation (exemplified by genes such as *ATP5MC3*, *ATP5MF*) and mitochondrial organization (*FIS1*, *TOMM7*), and that the downregulated genes were related to extracellular structure organization (*COL1A2*, *COL5A2*) and stemness maintenance (*KIT*, *ZIC3*) (Fig. 2A). These findings likely pinpoint mitochondrial modulation as the core mechanism causing dysregulation of hESC homeostasis upon RICTOR deficiency. Given that glycolysis regulates hESC self-renewal (Gu et al., 2016), we examined the changes in glycolysis in hESCs with RICTOR deficiency. Consistent with the aforementioned impaired hESC self-renewal upon RICTOR ablation, we observed decreased glycolysis in *RICTOR*^−/−^ hESCs (Fig. 2B). Subsequently, we evaluated the changes in the mitochondria between *RICTOR*^+/+^ and *RICTOR*^−/−^ hESCs and detected increases in mitochondrial number per cell and relative mitochondrial mass, suggesting higher mitochondrial content associated with RICTOR deficiency (Figs. 2C, 2D and S2C). By contrast, the ablation of RICTOR in hESCs caused disarranged cristae structure (Fig. 2C), increased mitochondrial ROS and decreased mitochondrial membrane potential (Fig. S2D and S2E), indicative of impaired mitochondrial fitness. Overall, although baseline mitochondrial respiration was comparable between *RICTOR*^+/+^ and *RICTOR*^−/−^ hESCs, the maximal respiratory ability was higher in *RICTOR*^−/−^ hESCs likely due to increased mitochondrial content (Fig. 2E). Altogether, these data indicate that RICTOR deficiency causes changes in glycolytic capacity and mitochondrial fitness in hESCs, likely contributing to impaired self-renewal capability.

**Figure 2:**
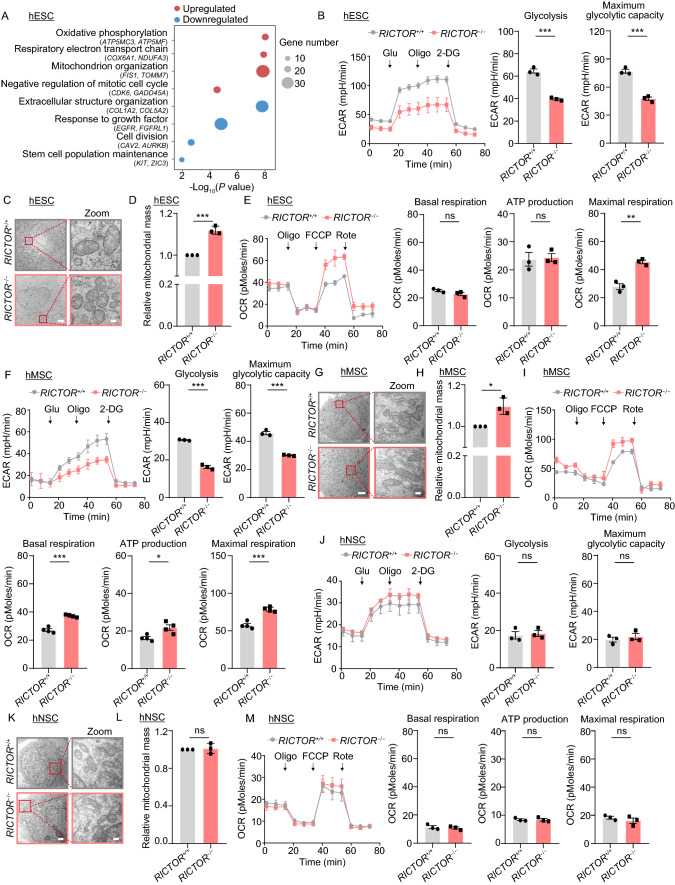
**RICTOR deficiency exerts differential effects on mitochondrial fitness in hESCs, hMSCs and hNSCs.** (A) GO and pathway enrichment analysis of differentially expressed genes in *RICTOR*^+/+^ and *RICTOR*^−/−^ hESCs. (B) Representative quantifications of extracellular acidification rates (ECAR) of *RICTOR*^+/+^ and *RICTOR*^−/−^ hESCs. Data are presented as mean ± SEM of three biological repeats. ***P < 0.001. (C) Representative transmission electron microscopy images of *RICTOR*^+/+^ and *RICTOR*^−/−^ hESCs. Scale bars, 2 μm and 200 nm (zoomed-in image). (D) Flow cytometric analysis of mitochondrial mass in hESCs. Data are presented as mean ± SEM of three independent experiments. ***P < 0.001. (E) Cellular oxygen consumption rates (OCR) of *RICTOR*^+/+^ and *RICTOR*^−/−^ hESCs in response to indicated mitochondrial modulators. Data are presented as mean ± SEM of three biological repeats. ns, not significant, **P < 0.01. (F) Representative quantifications of extracellular acidification rates (ECAR) of *RICTOR*^+/+^ and *RICTOR*^−/−^ hMSCs (passage 4). Data are presented as mean ± SEM of three biological repeats. ***P < 0.001. (G) Representative transmission electron microscopy images of *RICTOR*^+/+^ and *RICTOR*^−/−^ hMSCs (passage 4). Scale bars, 2 μm and 200 nm (zoomed-in image). (H) Flow cytometric analysis of mitochondrial mass in hMSCs (passage 4). Data are presented as mean ± SEM of three independent experiments. *P < 0.05. (I) Cellular oxygen consumption rates (OCR) of *RICTOR*^+/+^ and *RICTOR*^−/−^ hMSCs (passage 4) in response to indicated mitochondrial modulators. Data are presented as mean ± SEM of four biological repeats. *P < 0.05, ***P < 0.001. (J) Representative quantifications of extracellular acidification rates (ECAR) of *RICTOR*^+/+^ and *RICTOR*^−/−^ hNSCs (passage 5). Data are presented as mean ± SEM of three biological repeats. ns, not significant. (K) Representative transmission electron microscopy images of *RICTOR*^+/+^ and *RICTOR*^−/−^ hNSCs (passage 5). Scale bars, 1 μm and 200 nm (zoomed-in image). (L) Flow cytometric analysis of mitochondrial mass in hNSCs (passage 5). Data are presented as mean ± SEM of three independent experiments. ns, not significant. (M) Cellular oxygen consumption rates (OCR) of *RICTOR*^+/+^ and *RICTOR*^−/−^ hNSCs (passage 5) in response to indicated mitochondrial modulators. Data are presented as mean ± SEM of three biological repeats. ns, not significant

To determine the molecular mechanisms underlying RICTOR deficiency in human embryonic and adult stem cells, we performed RNA-seq analysis of hMSCs and hNSCs derived from *RICTOR*^+/+^ and *RICTOR*^−/−^ hESCs. In total, we identified 814 upregulated genes and 1,113 downregulated genes in hMSCs, and 1,056 upregulated genes and 530 downregulated genes in hNSCs (Fig. S2A and S2B). However, when we compared the DEGs identified in hMSCs and hNSCs to those of hESCs, we found that shared genes were relatively few, with only 14 upregulated and 29 downregulated genes in common (Fig. S2F). Of these downregulated were *BTF3* and *TOX*, previously reported to be related to stem cell maintenance and differentiation (de Jesus Domingues et al., 2016; Hu et al., 2019). By contrast, none of the overlapping genes were related to mitochondrial metabolism (Fig. S2F–S2H), suggesting that RICTOR deficiency impacts on cell type-specific mitochondrial regulatory mechanisms. Accordingly, we measured the changes in mitochondria and metabolism in RICTOR-deficient hMSCs and hNSCs. We found decreased glycolysis in *RICTOR*^−/−^ hMSCs (Fig. 2F), similarly as seen in *RICTOR*^−/−^ hESCs. Besides, RICTOR deficiency did not cause any detectable changes in mitochondrial cristae structure nor mitochondrial ROS levels between *RICTOR*^+/+^ and *RICTOR*^−/−^ hMSCs, but led to increased mitochondrial mass and membrane potential, likely indicative of enhanced mitochondrial respiratory capacity in *RICTOR*^−/−^ hMSCs (Figs. 2G, 2H, S2I and S2J). Indeed, we found that RICTOR deficiency increased both basal and maximal mitochondrial respiration in hMSCs (Fig. 2I). By comparison, in RICTOR-deficient hNSCs, we found RICTOR deficiency did not affect the glycolytic capacity (Fig. 2J), mitochondrial cristae structure, mitochondrial mass nor mitochondrial ROS production in hNSCs (Figs. 2K, 2L and S2K). In contrast to *RICTOR*^−/−^ hMSCs, we detected RICTOR deficiency led to decreased mitochondrial membrane potential in hNSCs (Fig. S2L). However, both basal and maximal mitochondrial respiration were normal in *RICTOR*^−/−^ hNSCs (Fig. 2M), suggesting that the mitochondrial dysregulation caused by RICTOR deficiency in hNSCs is tolerable. Altogether, our findings indicate that RICTOR is critical to the maintenance of mitochondrial fitness in hMSCs, but less important to mitochondrial function in hNSCs.

Here, for the first time, we systematically compared the effects of RICTOR deficiency in human embryonic stem cells and two adult stem cell types. By generating RICTOR-deficient hESCs and executing directed differentiation of these into hMSCs and hNSCs derivatives, we provided a valuable experimental platform for further studying biological roles of RICTOR in human embryonic and adult stem cell types. Our comprehensive analysis demonstrated that RICTOR deficiency adversely affected both self-renewal and differentiation abilities in all three human stem cell types. In addition, we offered novel insights into distinct mitochondrial and metabolic phenotypes caused by RICTOR deficiency in different human stem cells. Specifically, our data show that RICTOR was essential for glycolytic capacity and mitochondrial homeostatic maintenance in hESCs and hMSCs, but its absence led to tolerable mitochondrial defects without changing cell respiration in hNSCs. Our findings suggest that RICTOR maintains the stemness of hESCs and hMSCs likely associated with altered glycolysis and oxidative phosphorylation, and that mitochondrial respiration is largely independent of RICTOR in hNSCs. Overall, our data provide new knowledge about the differential roles of RICTOR in the homeostatic maintenance and mitochondrial regulation of different types of human stem cells.

Because the dysregulation of the mTOR pathway is a hallmark feature of diseases, including metabolic disorders, neurological disease and cardiovascular disease, mTOR is viewed with interest as a potential therapeutic target. To date, most studies on mTOR pathways has focused on mTORC1 via genetic manipulations or targeted pharmacological inhibitors such as rapamycin (Schreiber et al., 2019). By contrast, much less is known about the cellular consequences by interfering with mTORC2. In mice, depletion of Rictor, the unique core regulatory component of mTORC2, leads to embryonic growth arrest (Zhu et al., 2019) and impedes cardiac differentiation of embryonic stem cells (Zheng et al., 2017). In this study, we found that RICTOR deficiency impaired hESC self-renewal. In addition, our data revealed that RICTOR deficiency had varied impacts on differentiated derivatives and thus brought clarity to its pivotal roles in human stem cell maintenance and highlighting potential molecular and metabolic vulnerabilities in different human progenitor populations, which help us better understand the possible consequences of RICTOR inactivation in clinical cases.

Mitochondrial metabolism impacts on both the self-renewal and differentiation potentials of embryonic stem cells. Pluripotent stem cells have fewer mitochondria and produce energy mainly through glycolysis whereas differentiated cells rely primarily on oxidative phosphorylation and have increased mitochondrial mass. Additionally, attenuated mitochondrial activity negatively regulates cell proliferation and transcriptional activation of genes involved in early differentiation in both mouse and human ESCs (Mandal et al., 2011). Here, we found that RICTOR deficiency led to increased mitochondrial content and respiration in pluripotent hESCs and multipotent hMSCs, likely contributing to their compromised stem cell maintenance. Yet, in multipotent hNSCs, RICTOR deficiency only induced minimal, functionally tolerable changes in mitochondria, suggesting that impaired proliferation and differentiation capabilities of hNSCs lacking RICTOR are uncoupled from mitochondrial respiratory function. Notably, RICTOR has been implicated in multiple signaling pathways related to mitochondria. For instance, mTORC2-AKT-GSK3β signaling pathway participates in the maintenance of mitochondrial fitness and cellular metabolism (Bantug et al., 2018) and mTORC2-mitochondrial Connexin 43 signaling pathway directly modulates mitochondrial function (Wang et al., 2021b). Therefore, it awaits further investigations of the differential molecular mechanisms by which RICTOR deficiency interrupts mitochondrial homeostasis in hESCs and hMSCs, but not in hNSCs. Overall, our comparative data reveal RICTOR-dependent diverse mitochondria-regulatory and stemness maintenance mechanisms in human stem cell populations, possibly reflecting different metabolic needs in specific cell types or different degrees of pluripotency.

Taken together, our findings provide a molecular and functional basis for understanding how RICTOR regulates cellular and mitochondrial homeostasis in different human stem cells. As such, our data send a cautionary note for considering potential adverse effects resulting from treatment of various diseases using mTOR inhibitors, and offer important hints for the prognosis and even prevention of possible cellular and mitochondrial consequences in human stem cells in therapeutic strategies targeting RICTOR or other components of the mTOR pathway.

## Supplementary Information

Below is the link to the electronic supplementary material.

Supplementary file1 (PDF 2174 kb)

Supplementary file2 (XLSX 10 kb)

Supplementary file3 (XLSX 717 kb)
