# Correction to: mTORC2/RICTOR exerts differential levels of metabolic control in human embryonic, mesenchymal and neural stem cells

**DOI:** 10.1007/s13238-022-00917-3

**Published:** 2022-05-07

**Authors:** Qun Chu, Feifei Liu, Yifang He, Xiaoyu Jiang, Yusheng Cai, Zeming Wu, Kaowen Yan, Lingling Geng, Yichen Zhang, Huyi Feng, Kaixin Zhou, Si Wang, Weiqi Zhang, Guang-Hui Liu, Shuai Ma, Jing Qu, Moshi Song

**Affiliations:** 1 State Key Laboratory of Stem Cell and Reproductive Biology, Institute of Zoology, Chinese Academy of Sciences, Beijing, 100101 China; 2 State Key Laboratory of Membrane Biology, Institute of Zoology, Chinese Academy of Sciences, Beijing, 100101 China; 3 Institute for Stem Cell and Regeneration, Chinese Academy of Sciences, Beijing, 100101 China; 4 Beijing Institute for Stem Cell and Regenerative Medicine, Beijing, 100101 China; 5 Advanced Innovation Center for Human Brain Protection, National Clinical Research Center for Geriatric Disorders, Xuanwu Hospital Capital Medical University, Beijing, 100053 China; 6 CAS Key Laboratory of Genomic and Precision Medicine, Beijing Institute of Genomics, Chinese Academy of Sciences, Beijing, 100101 China; 7 China National Center for Bioinformation, Beijing, 100101 China; 8 University of Chinese Academy of Sciences, Beijing, 100049 China; 9 Aging Translational Medicine Center, International Center for Aging and Cancer, Xuanwu Hospital, Capital Medical University, Beijing, 100053 China; 10 Sino-Danish College, University of Chinese Academy of Sciences, Beijing, 101408 China; 11 Chongqing Renji Hospital, University of Chinese Academy of Sciences, Chongqing, 400062 China; 12 College of Life Sciences, University of Chinese Academy of Sciences, Beijing, 100049 China

## Correction to: Protein Cell 10.1007/s13238-021-00898-9

In this correction, the original supplementary files are replaced with the correct final version of the file.

The original article has been corrected.

