# Supplementary material for: mTORC2/RICTOR exerts differential levels of metabolic control in human embryonic, mesenchymal and neural stem cells
Source: Protein Cell. 2022 Jan 17;13(9):676–82. doi: 10.1007/s13238-021-00898-9 (PMC9233732; doi:10.1007/s13238-021-00898-9)
Supplement: Supplementary file 1 — Supplementary file1 (PDF 2174 kb) [file 13238_2021_898_MOESM1_ESM.pdf]

Supplemental Materials

Materials and Methods

Cell culture

RICTOR$^{+/+}$ and *RICTOR*$^{-/-}$ hESCs were cultured on feeder cells (mouse embryonic fibroblasts, MEFs) in hESC medium with DMEM/F12 (Invitrogen) supplemented with 20% Knockout Serum Replacement (Invitrogen), 2 mM GlutaMAX (Invitrogen), 0.1 mM non-essential amino acids (NEAA, Invitrogen), 1% penicillin/streptomycin (Invitrogen), 55 μM β-mercaptoethanol (Invitrogen), and 10 ng/mL FGF2 (Joint Protein Central) or on Matrigel (BD Biosciences) in mTeSR medium (STEMCELL Technology) in an incubator at 37°C with 5% CO_2.

Generation of *RICTOR*-knockout (*RICTOR*$^{-/-}$) hESCs

CRISPR/Cas9-mediated gene knockout was performed as previously described with minor modifications (Cheng et al., 2019). Specifically, the guide RNA (gRNA) (5'-AATATGGCGGCGATCGGCCGCGG-3') targeting the exon 1 of the *RICTOR* gene was cloned into the pCAG-mCherry-sgRNA vector (Addgene, #87110). H9 ESCs were cultured on Matrigel-coated plates for 4-5 days and then treated with ROCK inhibitor (Selleck Y-27632) overnight. The following day, 5×10^6 single cells were obtained via trypsinization and mixed with 100 μL Opti-MEM containing 14 μg pCAG-Cas9-2AGFP vectors (Addgene #87109) and 7 μg pCAG-mCherry-Rictor-gRNA vectors before the electroporation. After the electroporation, the cells were seeded on Matrigel-coated plates with mTeSR medium containing ROCK inhibitor. After 48 hr, cells were digested into single cells, and purified by fluorescence activated cell sorting (FACS) to obtain GFP and mCherry double-positive cells. Purified cells were seeded and cultured on MEF-coated plates. Clones were finally collected after about 10 days and subjected to the verification of gene editing success as well as downstream analyses.

Generation and characterization of hMSCs

Differentiation of hESCs into hMSCs was described previously (Zhang et al., 2019). Briefly, embryoid bodies (EBs) were formed from hESC clones in an ultralow attachment 6-well plate (Corning) in low-FGF2 hESC medium and were transferred to a Matrigel-coated plate in hMSC differentiation medium (hMSC culture medium supplemented with additional 9 ng/mL FGF2 and 5 ng/mL TGFβ (HumanZyme)). After 7 to 10 days, the cells became confluent and were further maintained in hMSC culture medium. hMSCs with triple positivity for CD73, CD90

and CD105 were sorted by flow cytometry and subjected to downstream analyses. The following antibodies were used: CD73-PE (BD Biosciences, 550257), CD90-FITC (BD Biosciences, 555595) and CD105-APC (Biolegend, 800508). The differentiative abilities of hMSCs were tested by differentiation into osteoblasts, chondrocytes and adipocytes as previously described (Zhang et al., 2019) and assessment with von Kossa staining (osteogenesis), toluidine blue staining (chondrogenesis) and Oil red O staining (adipogenesis), respectively.

Generation and characterization of hNSCs

hNSC differentiation from hESCs was conducted as previously described (Cheng et al., 2019). Briefly, hESCs were cultured on MEF feeders in neural induction medium-1 (neural stem cell maintenance medium supplemented with 1 μM CHIR99021, 1 μM SB431542, 2 μM dorsomorphin (Sigma) and 0.1 μM Compound E (EMD Chemicals Inc.) for two days, followed by neural induction medium-2 (neural induction medium-1 without dorsomorphin) for five days. The cells were subsequently cultured in Matrigel-coated plates in neural stem cell maintenance medium.

Neuronal differentiation from hNSCs

hNSCs were plated at a density of 3×10^4 cells per well in Matrigel-coated 24-well plates and cultured in neural stem cell maintenance medium for one to three days. The cells were then cultured in neuronal differentiation medium containing DMEM/F12, 1× N2, 1× B27, 200 μM Ascorbic acid (Sigma), 400 μM dbcAMP (Sigma), 10 ng/mL GDNF (Peprotech) and 10 ng/mL of BDNF (Peprotech) for two days before 20 μg/mL laminin (Sigma) was added to promote further differentiation.

Quantification of mRNA using real-time RT-PCR

Total RNAs were extracted from the cells with TRIzol Reagent (Thermo Fisher Scientific). Next, 2 μg of total RNA per sample was used for cDNA synthesis with the GoScript Reverse Transcription System (Promega) following the manufacturer’s instruction. RT-qPCR was performed on a QuantStudio™ 5 Real-Time PCR System (Applied Biosystems) using THUNDERBIRD SYBR qPCR Mix (TOYOBO). The relative expression of each gene was normalized to the GAPDH transcript. The primer sequences are listed in Supplementary Table S1.

Western blot analysis

The cells were lysed in RIPA buffer (Beyotime, P0013B) supplemented with a protease inhibitor cocktail (Roche, 4693159001) and a phosphatase inhibitor (Roche, 4906837001) and then centrifuged at 14,000 *g* for 15 min at 4°C. The supernatants were collected and quantified using a BCA protein quantification kit (Beijing Dingguo Changsheng Biotechnology, BCA-02), heat-denatured in 1× SDS loading buffer (Beijing Dingguo Changsheng Biotechnology, WB-0091) at 105°C for 5 min. The samples were then subjected to SDS-PAGE separation and electrotransferred onto 0.2 μm PVDF membrane (Millipore, ISEQ00010). The membrane was incubated with 5% (w/v) nonfat powdered milk (BBI Life Sciences, a600669-0250) for 1 hr at room temperature and then incubated with primary antibodies at 4°C overnight. HRP-conjugated secondary antibodies were used for incubation at room temperature for 1 hr before the visualization by using a ChemiDoc XRS+ system (Bio-Rad) and the data were analyzed with ImageJ. The primary antibodies used were as follows: RICTOR (Cell Signaling Technology, 2114), OCT3/4 (Santa Cruz, sc-5279), SOX2 (Santa Cruz, sc-17320), NANOG (Abcam, ab21624), AKT (Cell Signaling Technology, 4691), phospho-AKT (Ser473) (Cell Signaling Technology, 4060), β-Tubulin (Immunoway, YM3030), GAPDH (Santa Cruz, sc-69879), HRP-conjugated goat anti-mouse IgG (Jackson ImmunoResearch Laboratories, 115-035-003) and HRP-conjugated goat anti-rabbit IgG (Jackson ImmunoResearch Laboratories, 111-035-003).

Immunofluorescence staining

Cells were fixed with 4% paraformaldehyde for 20 min, permeabilized in 0.4% Triton X-100 in PBS for 20 min and incubated with 10% donkey serum in PBS for 20 min at room temperature. The cells were then incubated with primary antibodies at 4°C overnight followed by incubation with secondary antibodies as appropriate and Hoechst 33342 (Invitrogen) for 5 min at room temperature. The following antibodies were used: Ki67 (ZSGB-Bio, ZM-0166), PAX6 (Biolegend, 901301), SOX2 (Santa Cruz, sc17320), MAP2 (Sigma, M4403), β-tubulin III (TuJ1, Sigma, T2200), α-SMA (ZSGB-Bio, ZM-0003), FOXA2 (Cell Signaling Technology, 8186), Alexa 488 donkey anti-mouse IgG (Thermo Fisher Scientific, A21202), Alexa 568 donkey anti-rabbit IgG (Thermo Fisher Scientific, A10042) and Alexa 647 donkey anti-goat IgG (Thermo Fisher Scientific, A21447).

Alkaline phosphatase (AP) staining

An alkaline phosphatase detection kit (STEMGENT, 00-0055) was used for monitoring hESC undifferentiation through AP activity by immunocytochemistry staining. In brief, hESCs were fixed at room temperature for 3 min and washed with PBST (PBS containing 0.05% Tween-20). Fixed cells were then incubated in freshly prepared AP staining solution in the dark at room temperature for 15 min. The relative AP staining intensity was measured using ImageJ.

Teratoma formation assay

Teratoma formation assay was performed as previously described (Diao et al., 2021). In brief, 4×10^6 hESCs were collected and mixed with Matrigel:mTeSR (1: 4) solution. The mixture was then injected subcutaneously into the inguinal region of 6-week male NOD/SCID mice. The mice were monitored for teratoma formation and euthanized at about 10 weeks after the injection before the teratomas were collected for further analysis. The animal experiments performed in this study were approved by the Chinese Academy of Sciences Institutional Animal Care and Use Committee.

Clonal expansion assay

Briefly, 2×10^3 cells were seeded in 12-well plates and cultured for approximately 13 days. The cells were then fixed with 4% PFA for 30 min and stained with 0.2% crystal violet (Biohao, C0520) for 1 hr at room temperature and washed gently with running tap water. The relative cell density was measured using ImageJ.

Cell cycle analysis

For cell cycle analysis, 1×10^6 cells were collected and fixed in 75% ice-cold ethanol at -20°C overnight. Then, cells were washed twice with PBS and stained with 0.02 mg/mL propidium iodide and 0.2 mg/mL RNase at 37°C for 30 min. Cells were then analyzed by flow cytometry (BD LSR Fortessa).

Transmission electron microscopy

Transmission electron microscopy was performed to observe mitochondrial ultrastructure as previously reported (Diao et al., 2021). In brief, the cells were seeded onto Matrigel-coated microscope coverslips and fixed with 2.5% (vol/vol) glutaraldehyde at 4°C. The samples were dehydrated with graded alcohol and embedded in SPI-PON812 resin. Ultrathin sections with 70-nm thickness were obtained and then double-stained with uranyl acetate and lead citrate. Spirit Transmission Electron Microscope (FEI Company) was used to observe ultrastructure.

Mitochondrial numbers per cell were analyzed using ImageJ.

Measurement of mitochondrial mass, reactive oxygen species (ROS) and membrane potential

For mitochondrial mass measurement, cells were incubated with 10 μM of nonyl acridine orange (NAO, Thermo Fisher Scientific, A1372) for 10 min at 37°C and then measured by flow cytometry (BD LSRFortessa). For mitochondrial ROS level determination, cells were stained with MitoSOX Red (Thermo Fisher Scientific, M36008) for about 20 min at 37°C in the dark, and then analyzed by flow cytometry (BD LSRFortessa). For the assessment of mitochondrial membrane potential, cells were incubated with 1× JC-10 dye-loading solution (AAT Bioquest, 22801) at 37°C for 30 min, then measured by flow cytometry (BD LSRFortessa) and analyzed using FlowJo software. The ratio of fluorescence intensities Ex/Em = 490/590 (JC-10 aggregate emission) and 490/530 nm (JC-10 monomer emission) (FL590/FL530) were calculated to define mitochondrial membrane potentials.

Seahorse XF analysis

Cells were seeded onto a 96-well Seahorse XF96 extracellular analyzer plate at a density of 4×10^4 cells per well for hESCs, 3×10^4 cells per well for hMSCs and 4×10^4 cells per well for hNSCs. One day prior to the experiment, calibrant solution was added to XFe96 sensor cartridges and incubated overnight under 37°C without CO_2. On the day of the experiment, cells were washed twice and incubated for 45 min at 37°C with Seahorse assay media containing 10 mM Glucose, 1 mM sodium pyruvate and 2 mM L-Glutamine for mitochondrial stress protocol and 2 mM L-Glutamine only for glycolysis stress protocol. After 15 min of equilibration, for oxygen consumption rate (OCR) measurement, oligomycin, FCCP and rotenone/antimycin A were loaded into ports of the XF cartridge to achieve final concentrations of 1.5 μM, 1 μM and 0.5 μM in the reaction, respectively. For extracellular acidification rate (ECAR) analysis, glucose, oligomycin, 2-DG were loaded into ports of the XF cartridge to achieve final concentrations of 10 mM, 1 μM and 50 mM in the reaction, respectively. After the analysis, cells were fixed in 4% formaldehyde for 30 min and then incubated with Hoechst 33342 for 15 min at room temperature to perform nuclei count for data normalization for each well.

RNA-seq library construction and sequencing

Briefly, total RNAs were extracted using TRIzol Reagent and genomic DNA was removed

using a DNA-free Kit. Library preparation was achieved by using a NEBNext® UltraTM Directional RNA Library Prep Kit for Illumina (New England Biolabs). Quality control and sequencing on Illumina HiSeq X Ten platforms were performed by Novogene Bioinformatics Technology Co., Ltd.

RNA-seq data processing

For RNA-seq data, Trim Galore (version 0.5.0) was used to trim and filter reads. The filtered data were mapped to the UCSC human hg19 genome using HISAT2 (version 2.0.4) (Kim et al., 2015). Then, the mapped data for each gene were counted using HTSeq (version 0.11.0) (Anders et al., 2015). Differentially expressed genes (DEGs) were identified using the DESeq2 (version 1.26.0) (Love et al., 2014) with the cutoff Benjamini-Hochberg adjusted *P* value of 0.05 and the absolute fold change more than $\log_2(1.5)$. Enrichment analysis was performed using Metascape (http://metascape.org/gp/) (Zhou et al., 2019). The DEGs are listed in Supplementary Table S2.

CNV analysis

Raw data were trimmed using Trim Galore software. Trimmed data were mapped to the human reference genome (hg19) using Bowtie2 (version 2.2.9) (Langmead and Salzberg, 2012). Then, duplicate reads were removed using Picard (http://broadinstitute.github.io/picard) software. ReadCounter was used to calculate reads number for each 0.5 Mb and the R package HMMcopy (version 1.28.1) (Ha et al., 2012) was used to calculate CNVs.

Statistical analysis

Data are presented as the mean ± SEM. GraphPad Prism software was used to perform a two-tailed Student's *t*-test. Statistical significance was presented as $*P < 0.05$, $**P < 0.01$ and $***P < 0.001$.

Data availability

Whole genome sequencing and RNA-seq data generated in this study have been deposited in the Genome Sequence Archive in the National Genomics Data Center, Beijing Institute of Genomics (China National Center for Bioinformation) of the Chinese Academy of Sciences, under accession number HRA001236 that are publicly accessible at http://bigd.big.ac.cn/gsa-human. These data can also be accessed via an interactive user-friendly webtool at Aging Atlas (https://bigd.big.ac.cn/aging/index) (Aging Atlas Consortium, 2021).

References

Aging Atlas Consortium. (2021). Aging Atlas: a multi-omics database for aging biology. Nucleicacids research 49, D825-D830.

Anders, S., Pyl, P.T., and Huber, W. (2015). HTSeq--a Python framework to work with high-throughput sequencing data. Bioinformatics 31, 166-169.

Cheng, F., Wang, S., Song, M., Liu, Z., Liu, P., Wang, L., Wang, Y., Zhao, Q., Yan, K., Chan, P., *et al.* (2019). DJ-1 is dispensable for human stem cell homeostasis. Protein Cell 10, 846-853.

Diao, Z., Ji, Q., Wu, Z., Zhang, W., Cai, Y., Wang, Z., Hu, J., Liu, Z., Wang, Q., Bi, S., *et al.* (2021). SIRT3 consolidates heterochromatin and counteracts senescence. Nucleic Acids Res 49, 4203-4219.

Ha, G., Roth, A., Lai, D., Bashashati, A., Ding, J., Goya, R., Giuliany, R., Rosner, J., Oloumi, A., Shumansky, K., *et al.* (2012). Integrative analysis of genome-wide loss of heterozygosity and monoallelic expression at nucleotide resolution reveals disrupted pathways in triple-negative breast cancer. Genome Res 22, 1995-2007.

Kim, D., Langmead, B., and Salzberg, S.L. (2015). HISAT: a fast spliced aligner with low memory requirements. Nat Methods 12, 357-360.

Langmead, B., and Salzberg, S.L. (2012). Fast gapped-read alignment with Bowtie 2. Nat Methods 9, 357-359.

Love, M.I., Huber, W., and Anders, S. (2014). Moderated estimation of fold change and dispersion for RNA-seq data with DESeq2. Genome Biol 15, 550.

Zhang, X., Liu, Z., Liu, X., Wang, S., Zhang, Y., He, X., Sun, S., Ma, S., Shyh-Chang, N., Liu, F., *et al.* (2019). Telomere-dependent and telomere-independent roles of RAP1 in regulating human stem cell homeostasis. Protein Cell 10, 649-667.

Zhou, Y., Zhou, B., Pache, L., Chang, M., Khodabakhshi, A.H., Tanaseichuk, O., Benner, C., and Chanda, S.K. (2019). Metascape provides a biologist-oriented resource for the analysis of systems-level datasets. Nat Commun 10, 1523.

Figure S1

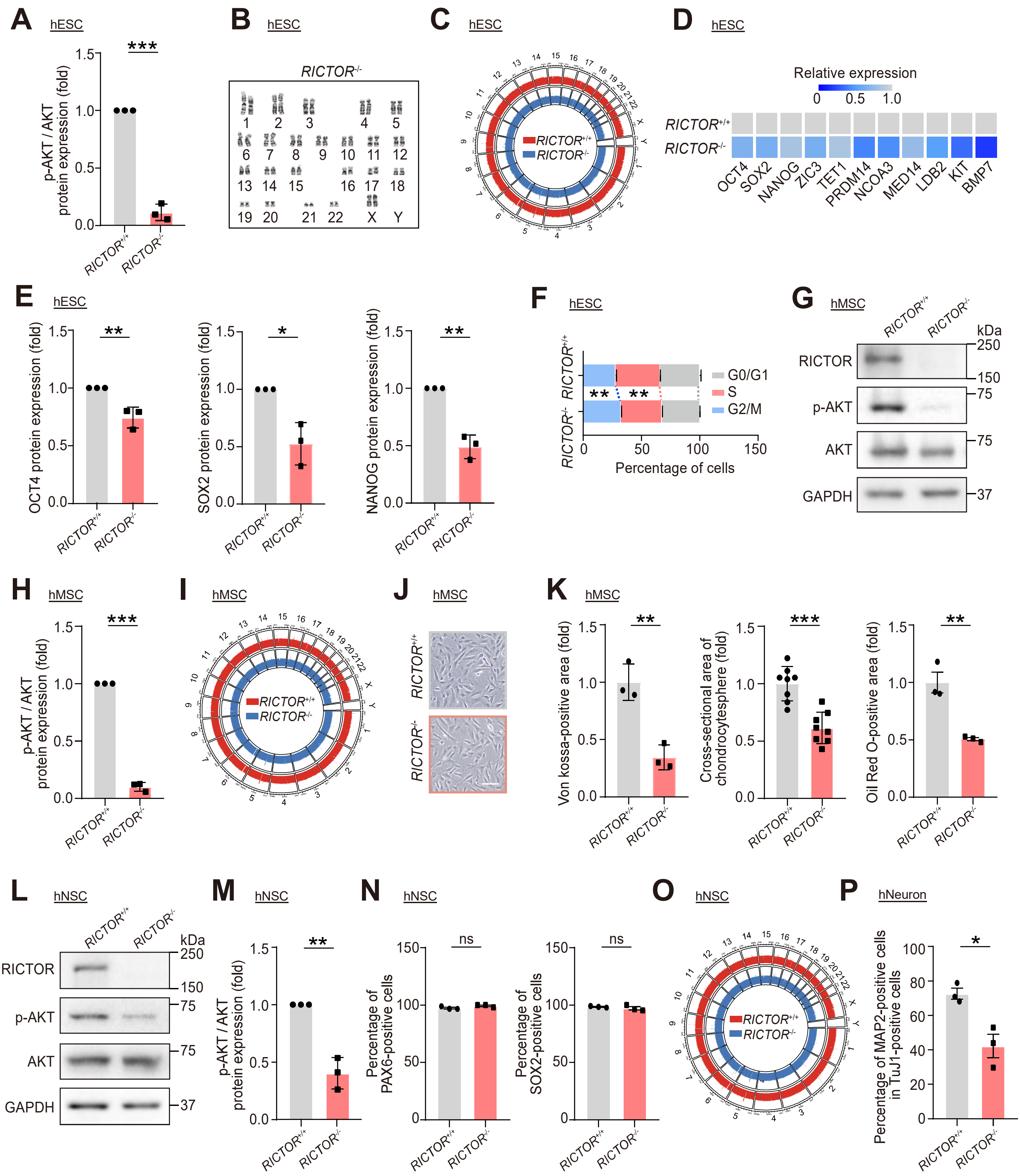

A hESC
p-AKT / AKT protein expression (fold)
***
RICTOR+/+
RICTOR-/-
B hESC
RICTOR-/-
C hESC
D hESC
Relative expression
0 0.5 1.0
OCT4 SOX2 NANOG ZIC3 TET1 PRDM14 NCOA3 MED14 LDB2 KIT BMP7
E hESC
OCT4 protein expression (fold)
SOX2 protein expression (fold)
NANOG protein expression (fold)
F hESC
G0/G1
S
G2/M
Percentage of cells
G hMSC
RICTOR
p-AKT
AKT
GAPDH
kDa
H hMSC
I hMSC
J hMSC
K hMSC
Von kossa-positive area (fold)
Cross-sectional area of chondrocytesphere (fold)
Oil Red O-positive area (fold)
L hNSC
M hNSC
N hNSC
Percentage of PAX6-positive cells
Percentage of SOX2-positive cells
ns
O hNSC
P hNeuron
Percentage of MAP2-positive cells in TuJ1-positive cells

Figure S2

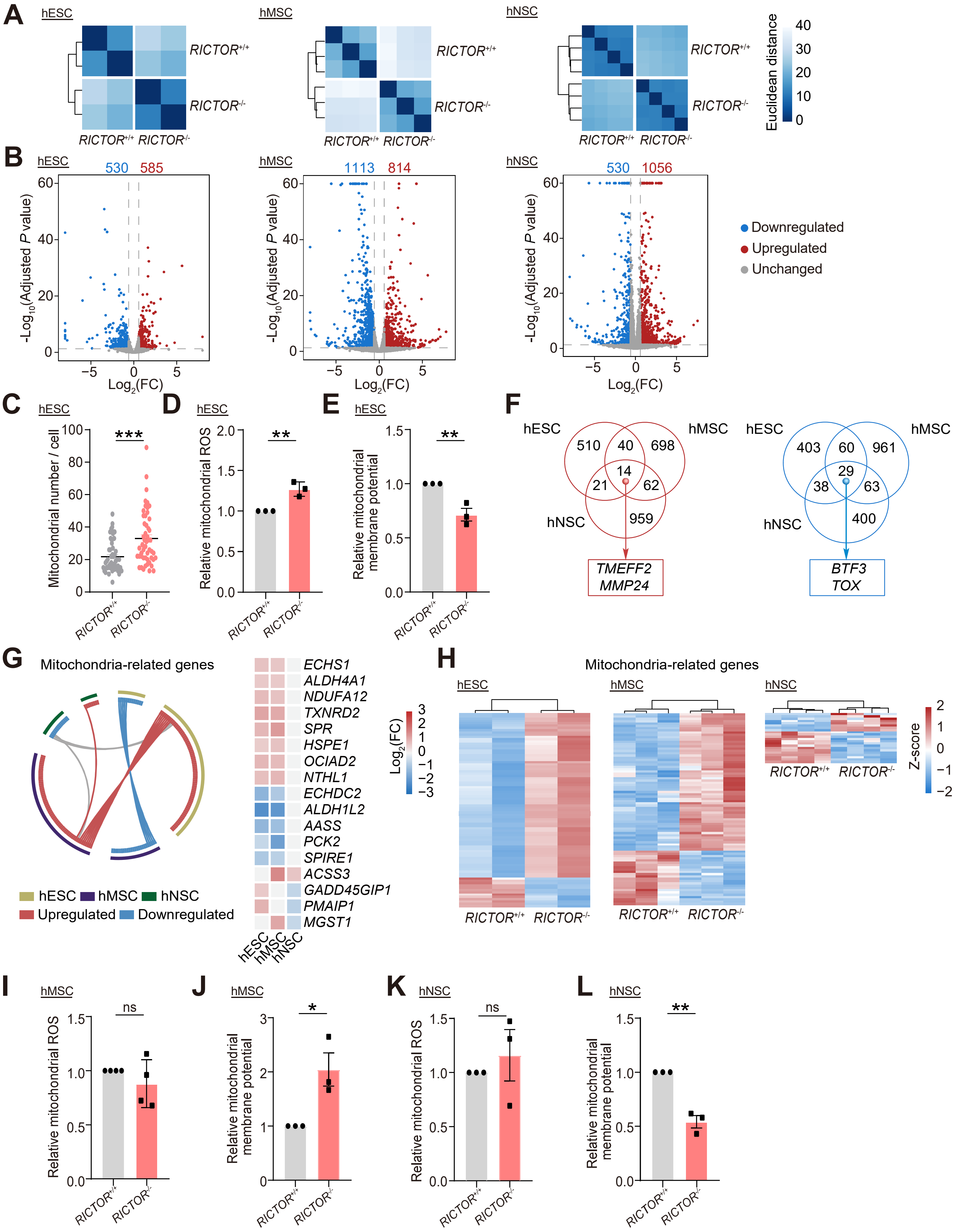

A
hESC
hMSC
hNSC
RICTOR+/+
RICTOR-/-
Euclidean distance
B
530 585
1113 814
530 1056
-Log10(Adjusted P value)
Log2(FC)
Downregulated
Upregulated
Unchanged
C
hESC
Mitochondrial number / cell
***
D
Relative mitochondrial ROS
**
E
Relative mitochondrial membrane potential
**
F
510 40 698 14 21 62 959
TMEFF2
MMP24
403 60 961 29 38 63 400
BTF3
TOX
G
Mitochondria-related genes
hESC hMSC hNSC
Upregulated Downregulated
ECHS1
ALDH4A1
NDUFA12
TXNRD2
SPR
HSPE1
OCIAD2
NTHL1
ECHDC2
ALDH1L2
AASS
PCK2
SPIRE1
ACSS3
GADD45GIP1
PMAIP1
MGST1
Log2(FC)
H
Mitochondria-related genes
Z-score
I
hMSC
ns
J
*
K
ns
L
**

Supplemental Figure Legends

Figure S1. Phase-contrast images, quantifications of multiple RNAs and proteins, and functional analyses of RICTOR-deficient hESCs, hMSCs and hNSCs.

(A) Quantification of normalized phosphorylated AKT Ser473 levels to total AKT expression in $RICTOR^{+/+}$ and $RICTOR^{-/-}$ hESCs. Data are presented as mean ± SEM of three independent experiments. $^{***}P < 0.001$. These quantitative results are related to Fig 1C. **(B)** Karyotype analysis of $RICTOR^{-/-}$ hESCs. **(C)** Genome-wide analysis of copy number variations (CNVs) by whole genome sequencing in $RICTOR^{+/+}$ and $RICTOR^{-/-}$ hESCs. **(D)** Heatmap showing the mRNA expression of the pluripotency markers *OCT4*, *SOX2*, *NANOG* and a few other genes related to stem cell population maintenance in $RICTOR^{+/+}$ and $RICTOR^{-/-}$ hESCs. **(E)** Western blot analysis of protein expression of hESC pluripotency markers OCT4, SOX2 and NANOG in $RICTOR^{+/+}$ and $RICTOR^{-/-}$ hESCs. β-tubulin was used as the loading control. Data are presented as mean ± SEM of three independent experiments. $^{**}P < 0.01$, $^{*}P < 0.05$. These quantitative results are related to Fig 1E. **(F)** Cell cycle analysis of $RICTOR^{+/+}$ and $RICTOR^{-/-}$ hESCs. Data are presented as mean ± SEM of three biological repeats. $^{**}P < 0.01$. **(G)** Western blot analysis of RICTOR, phosphorylated AKT Ser473 level and total AKT expression in $RICTOR^{+/+}$ and $RICTOR^{-/-}$ hMSCs. GAPDH was used as the loading control. **(H)** Quantification of normalized phosphorylated AKT Ser473 levels to total AKT expression in $RICTOR^{+/+}$ and $RICTOR^{-/-}$ hMSCs. Data are presented as mean ± SEM of three independent experiments. $^{***}P < 0.001$. **(I)** Genome-wide analysis of copy number variations (CNVs) by whole genome sequencing in $RICTOR^{+/+}$ and $RICTOR^{-/-}$ hMSCs. **(J)** Representative phase-contrast images of $RICTOR^{+/+}$ and $RICTOR^{-/-}$ hMSC colonies. Scale bar, 100 μm. **(K)** Quantifications of the multilineage differentiation potentials of hMSCs. Left, osteogenesis of $RICTOR^{+/+}$ and $RICTOR^{-/-}$ hMSCs evaluated by von Kossa staining. Data are presented as mean ± SEM of three biological repeats. $^{**}P < 0.01$. Middle, chondrogenesis of $RICTOR^{+/+}$ and $RICTOR^{-/-}$ hMSCs evaluated by Toluidine blue staining. Data are presented as mean ± SEM of eight biological repeats. $^{***}P < 0.001$. Right, adipogenesis of $RICTOR^{+/+}$ and $RICTOR^{-/-}$ hMSCs evaluated by Oil Red O staining. Data are presented as mean ± SEM of three biological repeats. $^{**}P < 0.01$. These quantitative results are related to Fig 1M. **(L)** Western blot analysis of RICTOR, phosphorylated AKT Ser473 level and total AKT expression in $RICTOR^{+/+}$ and

RICTOR$^{-/-}$ hNSCs. GAPDH was used as the loading control. **(M)** Quantification of normalized phosphorylated AKT Ser473 levels to total AKT expression in *RICTOR*$^{+/+}$ and *RICTOR*$^{-/-}$ hNSCs. Data are presented as mean ± SEM of three independent experiments. $^{**}P < 0.01$. **(N)** Quantifications of immunofluorescence staining for hNSC-specific markers PAX6 and SOX2 in *RICTOR*$^{+/+}$ and *RICTOR*$^{-/-}$ of hNSCs. Data are presented as mean ± SEM of three biological repeats. ns, not significant. These quantitative results are related to Fig 1N. **(O)** Genome-wide analysis of copy number variations (CNVs) by whole genome sequencing in *RICTOR*$^{+/+}$ and *RICTOR*$^{-/-}$ hNSCs. (P) Quantifications of the percentages of MAP2-positive cells in TuJ1-positive cells in *RICTOR*$^{+/+}$ and *RICTOR*$^{-/-}$ hNeurons. Data are presented as mean ± SEM of three biological repeats. $^{*}P < 0.05$. These quantitative results are related to Fig 1Q.

Figure S2. RNA sequencing analysis and functional analysis of RICTOR-deficient hESCs, hMSCs and hNSCs.

(A) Heatmap showing the Euclidean distance between RNA-seq replicates of *RICTOR*$^{+/+}$ and *RICTOR*$^{-/-}$ hESCs, hMSCs and hNSCs. **(B)** Volcano plot of differentially expressed genes in *RICTOR*$^{+/+}$ and *RICTOR*$^{-/-}$ hESCs, hMSCs and hNSCs. **(C)** Transmission electron microscopy analysis of mitochondrial number in *RICTOR*$^{+/+}$ and *RICTOR*$^{-/-}$ hESCs. Data are presented as mean ± SEM of 50 cells. $^{***}P < 0.001$. These quantitative results are related to Fig 2C. **(D)** Flow cytometric analysis of mitochondrial ROS in *RICTOR*$^{+/+}$ and *RICTOR*$^{-/-}$ hESCs. Data are presented as mean ± SEM of three independent experiments. $^{**}P < 0.01$. **(E)** Flow cytometric analysis of relative mitochondrial membrane potential in *RICTOR*$^{+/+}$ and *RICTOR*$^{-/-}$ hESCs. Data are presented as mean ± SEM of three independent experiments. $^{**}P < 0.01$. **(F)** Shared upregulated and downregulated genes in *RICTOR*$^{+/+}$ and *RICTOR*$^{-/-}$ hESCs, hMSCs and hNSCs. Red indicates upregulated gene and blue indicates downregulated gene. **(G)** Shared mitochondria-related genes in *RICTOR*$^{+/+}$ and *RICTOR*$^{-/-}$ hESCs, hMSCs and hNSCs. **(H)** Heatmaps showing the expression levels of mitochondria-related genes in *RICTOR*$^{+/+}$ and *RICTOR*$^{-/-}$ hESCs, hMSCs and hNSCs. **(I)** Flow cytometric analysis of mitochondrial ROS in *RICTOR*$^{+/+}$ and *RICTOR*$^{-/-}$ hMSCs. Data are presented as mean ± SEM of four independent experiments. ns, not significant. **(J)** Flow cytometric analysis of relative mitochondrial membrane potential in *RICTOR*$^{+/+}$ and *RICTOR*$^{-/-}$ hMSCs. Data are presented as mean ± SEM

of three independent experiments. *$P < 0.05$. **(K)** Flow cytometric analysis of mitochondrial ROS in *RICTOR*$^{+/+}$ and *RICTOR*$^{-/-}$ hNSCs. Data are presented as mean ± SEM of three independent experiments. ns, not significant. **(L)** Flow cytometric analysis of relative mitochondrial membrane potential in *RICTOR*$^{+/+}$ and *RICTOR*$^{-/-}$ hNSCs. Data are presented as mean ± SEM of three independent experiments. **$P < 0.01$.

Supplemental Table Legends

Table S1. RNA primers for qRT-PCR assay.

Table S2. DEGs in *RICTOR*$^{-/-}$ versus *RICTOR*$^{+/+}$ hESCs, hMSCs and hNSCs.
